# SSR and IRAP-based genetic diversity analysis for core collection of *Idesia polycarpa*

**DOI:** 10.1186/s12870-026-09068-7

**Published:** 2026-05-28

**Authors:** Huang Yang, Junrong Wang, Fuhua Fan

**Affiliations:** 1 Institute for Forest Resources and Environment of Guizhou Province/Guizhou Key Laboratory of Forest Cultivation in Plateau Mountain, Guizhou University, Guiyang, Guizhou 550025 China; 2 College of Forestry, Guizhou University, Guiyang, Guizhou 550025 China; 3 Xingyi Forestry Bureau, Xingyi, Guizhou 562400 China

**Keywords:** Woody oil crop, *Salicaceae*, Molecular markers, Population structure, Germplasm conservation

## Abstract

*Idesia polycarpa*, a promising woody oil crop, has abundant germplasm resources in Guizhou Province. To promote effective conservation and utilization, a core collection was established. The genetic diversity of 120 accessions from seven populations was evaluated using 42 polymorphic primers (18 SSRs, 24 IRAPs). The markers showed high polymorphism (PPL: 100%). IRAPs outperformed SSRs in the number of loci per primer (17.76 vs. 9.56), *Ne* (1.43 vs. 1.31), *H* (0.27 vs. 0.20), *I* (0.61 vs. 0.46), and PIC (0.71 vs. 0.89). The Mantel test indicated that both markers provided complementary information (*r* = 0.424, *p* < 0.001). Genetic diversity varied significantly among populations, with the Guizhou northwest region (LPS) the most diverse and the Guizhou southeast region (QDN) the least. Analysis showed moderate to low genetic differentiation (*Gst* = 0.126) and moderate gene flow (*Nm* = 1.73). UPGMA, PCoA, and STRUCTURE analyses revealed a distinct genetic structure with some local admixture. A core collection of 30 accessions was successfully established, retaining 96.7% of the total alleles. DNA fingerprints using IRAP primers (RT19 + RT34) achieved 100% discrimination among individuals. This study provides valuable genetic insights and technical support for the conservation, breeding, and sustainable use of *Idesia polycarpa*.

**Supplementary Information:**

The online version contains supplementary material available at 10.1186/s12870-026-09068-7.

## Introduction

*Idesia polycarpa* (hereafter *I. polycarpa*), commonly known as the oil grape, is the sole species in the genus *Idesia* (*Salicaceae*) [1]. It is native to East Asia, with natural ranges across the Korean Peninsula, Japan, and southern China [2]. As a high-value woody oilseed tree, it produces air-dried fruits containing up to 45% (w/w) oil—comparable to oil palm (*Elaeis guineensis*, ~ 50%) and higher than soybean (*Glycine max*, ~ 20%) and rapeseed (*Brassica napus*, ~ 40%) [3–9]. A single mature tree can produce 1.5–2.5 kg of crude oil, translating to an estimated yield of 2.25–3.75 tons per hectare—surpassing the average per-hectare yield of sunflower (*Helianthus annuus*, 0.9–1.2 tons) and approaching that of oil palm (3–4 tons) [3, 10]. A key feature of *I. polycarpa* oil is its exceptionally high linoleic acid (LA; C18:2) content, which significantly exceeds that of traditional woody oilseed crops—including oil palm, olive (*Olea europaea*), and *Camellia oleifera* [11]. Linoleic acid is an essential omega-6 fatty acid with well-known health benefits: clinical and epidemiological studies demonstrate that adequate dietary intake of LA supports improved lipid metabolism. Specifically, LA helps lower low-density lipoprotein cholesterol (LDL-C), raise high-density lipoprotein cholesterol (HDL-C), and supports glucose–insulin regulation and a healthy body weight [12–14]. In addition to LA, the oil contains several bioactive compounds of nutritional and functional importance, including squalene, volatile terpenoids, tocopherols (vitamin E), and phenolic compounds. Due to its safe profile and established health benefits, *I. polycarpa* oil was officially approved as a new food ingredient by China’s National Health Commission in 2020 [11].

As an emerging woody oilseed species, *I. polycarpa* faces challenges in both conservation and utilization. Its dioecious, insect-pollinated reproductive system promotes outcrossing and gene flow, but habitat fragmentation can disrupt these processes and accelerate genetic erosion. Establishing a core collection is therefore essential for capturing maximum genetic diversity and structural variation with a minimal set of accessions [15]. A thorough understanding of genetic diversity and population structure is crucial for the efficient collection, conservation, and use of *I. polycarpa* germplasm. Research on core collections has already covered various woody oil crops, such as *Camellia oleifera*, *Olea europaea*, *Elaeis guineensis*, *Juglans regia*, and *Xanthoceras sorbifolium* [16–20], with ongoing efforts for *I. polycarpa*. Guizhou Province—a core area of wild diversity for this species—is facing increasing pressures from habitat loss and over-harvesting, along with a lack of systematic conservation efforts. Given the abundance of wild germplasm in Guizhou, coupled with critical challenges such as gaps in genetic information and unclear genetic relationships, establishing a core collection for *I. polycarpa* would not only improve genetic resource management but also support breeding programs.

Currently, the primary data used to construct a core collection of germplasm resources are based on morphological and molecular markers [21]. However, developing a core collection based on morphological traits is susceptible to variation due to environmental factors and developmental stage. In contrast, molecular marker technology, by elucidating the genetic basis, offers greater stability and accuracy. Over the past decade, most studies on plant core collections have used molecular markers to construct them [22]. Two molecular marker types, the Simple Sequence Repeats (SSRs) and Inter-Retrotransposon Amplified Polymorphism (IRAP), have been widely applied due to their advantages of high polymorphism, abundance, operational simplicity, and low cost, making them suitable for genetic diversity analysis in a variety of plant species. In terms of germplasm selection, both markers have been effectively used for cultivar identification and for assembling germplasm collections [23–26].

This study aims to develop SSR and IRAP molecular markers to analyze the genetic diversity of *I. polycarpa* in Guizhou Province and to establish a core collection. The findings are expected to provide a scientific basis for selecting elite cultivars, identifying germplasm, and innovative utilization of this tree species.

## Materials and methods

### Plant materials and DNA extraction

**Fig. 1:**
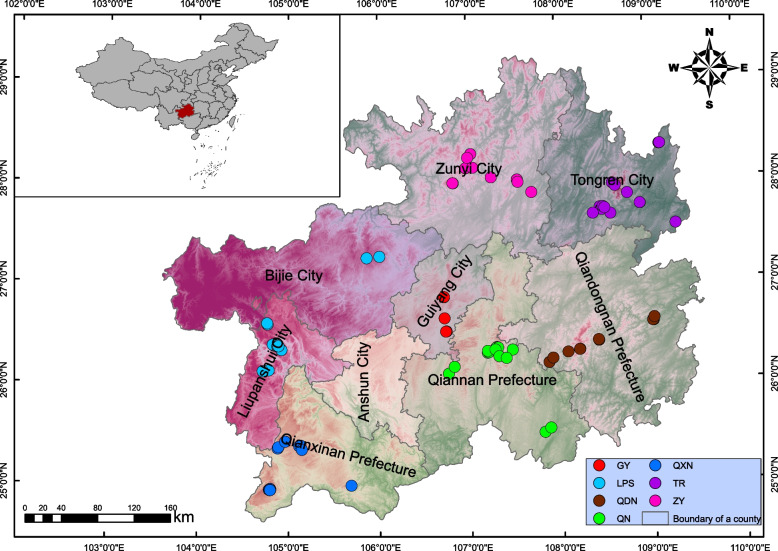
Distribution map of wild *I. polycarpa* germplasm resources in Guizhou Province. GY, Guizhou Central Region, including Guiyang City and Xiuwen County; QN, Guizhou Southern Region, including Duyun City, Guiding County, Huishui County, and Liping County; QXN, Guizhou Southwest Region, including Xingyi City, Xingren City, Pu’an County, and Chehen County; LPS, Guizhou Northwest Region, including Liupanshui City and Dafang County; ZY, Guizhou Northern Region, including Meitan County, Suiran County, and Huishan District; TR, Tongren City, including Jiangkou County, Yinjiang County, Songtao County, and Wanshan District; QDN, Guizhou Southeast Region, including Danzai County, Jianhe County, Jinping County, and Leishan County; hereinafter referred to as GY, QN, LPS, ZY, TR, QDN, respectively

For this study, we obtained permission from the local forestry department to collect samples of *I. polycarpa*. Wild individuals of this species are patchily distributed in Guizhou Province. After extensive field surveys covering most counties, we found that large, continuous natural populations are rare; in many counties, only one or a few isolated trees were found. Therefore, rather than defining populations based on natural distribution patches (which are often indistinct or too small), we grouped samples by administrative regions (prefectures/cities) as a practical proxy for geographic origin. Sampling was conducted across seven administrative regions, spanning the eastern, southern, western, and northern directions from the provincial capital. A total of 120 individuals were collected, each representing one germplasm accession (Fig. 1, Supplementary Table S1). To avoid sampling closely related or clonal individuals (e.g., siblings or ramets) at the local scale, the minimum distance between sampled trees within the same county or on the same slope was set to > 50 m. Distances between administrative regions are naturally much larger (tens to hundreds of kilometers).

Sample collection followed sterile procedures, and materials were immediately stored at − 80 °C for later analysis. Genomic DNA was extracted from young leaves using the DP320 Plant Genomic DNA Extraction Kit (Tiangen Biochemical Technology (Beijing) Co., Ltd.). DNA quality was checked with 1% agarose gel electrophoresis, and purity and concentration were measured using a UV–Vis spectrophotometer. Qualified DNA samples were diluted to 20 ng/μL in TE Buffer and stored at − 20 °C until use.

### Primer selection and PCR amplification

We developed SSR and IRAP molecular markers based on the *I. polycarpa* genome. A total of 42 primer pairs were initially screened, including 18 SSR primer pairs and 24 IRAP primers (Supplementary Table S2) [27]. These primers were used to analyze the genetic diversity of all samples. The PCR reaction system, amplification conditions, electrophoresis separation conditions, and band scoring methods are detailed in Supplementary Table S3.

### Analysis of genetic diversity parameters

Based on the electrophoresis results, a binary (0/1) matrix was created, and genetic diversity parameters were calculated using R version 4.2.3. These parameters include the number of alleles (*Na*); effective number of alleles (*Ne*) = 1/Σ(pi^2^) [28]; Nei’s gene diversity (*H*) = 1 – Σ(pi^2^) [29]; and Shannon’s information index (*I*) = – Σ(pi·log_2_(pi)), where pi represents the frequency of the *i*-th allele [30]; Polymorphism information content (PIC) was computed using the software PIC_CALC version 0.6.

### Cluster analysis and population structure analysis

Cluster analysis, Principal Coordinates Analysis (PCoA), and Mantel test [31] were conducted using the “vegan [32],” “ggplot2 [33],” “ape [34],” “dplyr [35],” “tidyr [36],” “ggrepel [37],” “scales [38],” “extrafont [39],” and “phangorn [40]” packages in R version 4.2.3. Population structure was evaluated using STRUCTURE, and the optimal number of populations was determined. The overall structure was inferred using a Bayesian model, with the maximum-likelihood value calculated for each K. A higher maximum-likelihood value indicates that K is closer to the actual structure [41]. The burn-in period and MCMC iterations were both set to 10,000. The grouping value (K) for subgroups ranged from 2 to 10, with each K simulated in triplicate. Subsequently, the data were uploaded to the Structure Harvester website (http://taylor0.biology.ucla.edu/structureHarvester/) to identify the most appropriate K based on the ΔK method.

### Core collection development and genetic diversity assessment

PowerCore 1.0 software was used to select minimally redundant and most representative subsets of germplasm from the original collection by randomly sampling at proportions of 5%, 10%, 15%, 20%, 25%, and 30% [42]. The genetic diversity parameters of the entire collection and the core collections were compared using one-way analysis of variance (ANOVA) in SPSS 25.

## Results

### Analysis of molecular marker polymorphism

In this study, a total of 596 alleles were identified, with a 100% polymorphism rate across all bands, indicating that the chosen markers have high discriminative power in *I. polycarpa* (Supplementary Table S4). SSR markers produced 172 alleles, averaging 9.56 polymorphic loci per primer. Among these, SSR2 generated the most polymorphic loci (18), while SSR20 and SSR38 each produced the fewest (5). For IRAP markers, a total of 424 fragments were amplified, with an average of 17.67 polymorphic loci per primer. RT34 generated the most polymorphic loci (21), whereas RT7 produced the fewest (14).

Among the SSR primers, SSR38 showed the highest values for *Ne* (1.53), *I* (0.33), and *H* (0.72) (Supplementary Table S4). The average *Ne*, *H*, and *I* across all SSR primers were 1.31, 0.20, and 0.46, respectively. The PIC values for SSR markers ranged from 0.54 (SSR9) to 0.90 (SSR2), with a mean of 0.71. For IRAP primers, RT33 exhibited the highest *Ne* (1.57), *H* (0.34), and* I* (0.74). The mean *Ne*, *H*, and *I* for all IRAP primers were 1.43, 0.27, and 0.61, respectively. The PIC values for IRAP markers ranged from 0.82 (RT11) to 0.93 (RT34), with a mean of 0.89.

Overall, IRAP markers showed slightly higher values than SSR markers for *Ne*, *H*, *I* and PIC. Both marker systems proved effective in revealing the genetic diversity of *I. polycarpa* germplasm.

### Analysis of concordance between the two molecular markers

**Fig. 2:**
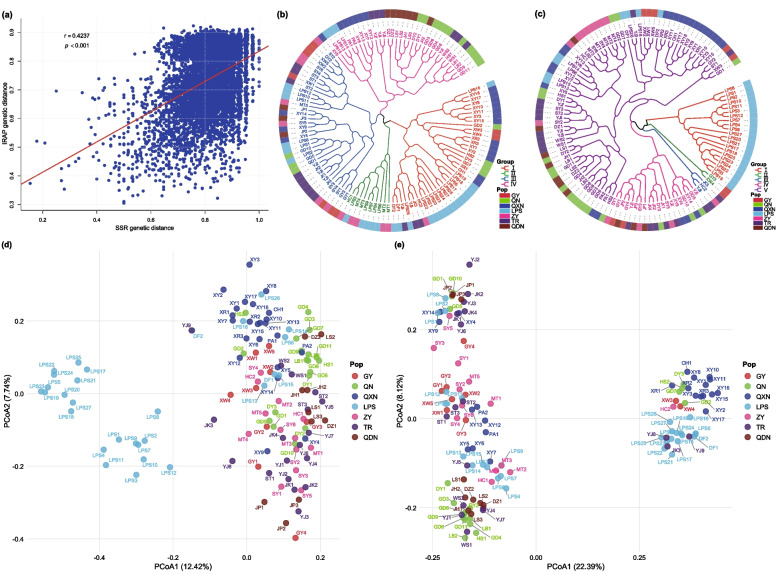
Genetic diversity of 120 *I. polycarpa* accessions based on SSR and IRAP markers. **a** Mantel test correlation between SSR and IRAP genetic distances (*r* = 0.4237, *p* < 0.001). **b** Dendrogram of 120 accessions based on SSR markers, constructed using the UPGMA method and the Jaccard similarity coefficient. **c** Dendrogram of 120 accessions based on IRAP markers (UPGMA, Jaccard coefficient). **d** Principal coordinate analysis (PCoA) of SSR markers. PCo1 explains 12.42% of the variance, and PCo2 explains 7.74% of the variance. **e** Principal coordinate analysis (PCoA) of IRAP markers. PCo1 explains 22.39% of the variance, and PCo2 explains 8.12% of the variance

Mantel test results (Fig. 2a) showed a significant correlation (*r* = 0.424, *p* < 0.001) between the genetic distance matrices derived from SSR and IRAP markers. This demonstrates some agreement between the two marker systems in revealing the genetic structure of *I. polycarpa*, while also highlighting notable differences.

To further explore the similarities and differences between the two marker types, cluster analyses were conducted on 120 germplasm accessions using SSR and IRAP data, respectively. The clustering based on SSR markers divided the samples into five groups (Fig. 2b, Supplementary Table S5). Group I included only 21 accessions from the LPS region, all sharing a common trait—pubescence on the underside of the leaf—a feature not found in accessions from other groups (Supplementary Fig. S1). This indicates that SSR markers effectively differentiate this morphologically distinct group. The other groups (II–V) mainly contained germplasm from different geographic origins, indicating some geographic mixing. For example, Group V included 74 accessions from various regions, such as GY, QN, QXN, LPS, ZY, TR, and QDN, suggesting that SSR markers can identify broad genetic similarities across diverse materials.

In contrast, clustering based on IRAP markers grouped the materials into four categories (Fig. 2d, Supplementary Table S6), with a pattern that differed significantly from that of SSR. The LPS accessions with pubescent leaves, which formed a distinct cluster (Group I) in the SSR analysis, were spread across the first three groups in the IRAP results. Meanwhile, the genetically mixed Group V identified by SSR was divided into Groups I, III, and IV by IRAP. Notably, no IRAP group consisted entirely of accessions of a single morphological type, unlike Group I in the SSR clustering.

To examine whether an alternative approach could corroborate the dendrogram analysis, Principal Coordinate Analysis (PCoA) was conducted. The SSR-based PCoA distinctly separated the pubescent-leaf LPS accessions from others, demonstrating its high effectiveness in revealing genetic structures linked to local adaptation or specific traits (Fig. 2c). Although the IRAP-based PCoA also distinctly separated 38 accessions from regions such as LPS and QXN from the remaining samples, this separation was not directly associated with a specific localized phenotype (Fig. 2e).

To gain a more thorough and precise understanding of its genetic diversity and population structure, we used both SSR and IRAP marker systems in our study to develop a core collection of *I. polycarpa* germplasm.

### Analysis of genetic diversity across various *I. polycarpa* populations

Raw genetic diversity indices (*H* and *I*) were strongly correlated with sample size (*r* = 0.909, *p* < 0.01; Table 1): LPS (*n* = 29) had the highest raw values (*H* = 0.239, *I* = 0.371), whereas GY (*n* = 9) and QDN (*n* = 10) had the lowest (*H* = 0.182 and 0.157, respectively). To correct this sampling bias, we rarefied to a common sample size (*n* = 9) with 1000 bootstrap replicates.

**Table 1:** Genetic diversity among different *I. polycarpa* populations

Pop	N	Raw *H*	Raw *I*	Rarefied *H* (95% CI)	Rarefied *I* (95% CI)
LPS	29	0.239	0.371	0.220 (0.197–0.236)	0.334 (0.299–0.357)
QXN	23	0.211	0.324	0.196 (0.174–0.208)	0.295 (0.265–0.314)
TR	18	0.218	0.336	0.206 (0.179–0.224)	0.311 (0.268–0.336)
QN	18	0.205	0.319	0.193 (0.163–0.212)	0.294 (0.246–0.321)
ZY	13	0.188	0.289	0.181 (0.161–0.193)	0.274 (0.245–0.293)
QDN	10	0.157	0.239	0.155 (0.149–0.160)	0.235 (0.228–0.242)
GY	9	0.182	0.276	0.182 (0.182–0.182)*	0.276 (0.276–0.276)*

^*^For GY (*n* = 9), rarefaction to *n* = 9 always samples all individuals, resulting in no confidence interval range. *H* = Nei’s gene diversity; *I* = Shannon’s information index; CI = confidence interval from 1000 bootstrap replicates

After rarefaction, the correlation between sample size and expected diversity (*H*_rare) was eliminated (*r* = 0.18,* p* > 0.05). Rarefaction curves showed diminishing returns with increasing sample size, and their slopes approached zero for most populations (Supplementary Figure S2), suggesting that sampling was sufficient to capture most within‑population diversity.

LPS retained significantly higher expected diversity (*H*_rare = 0.220, 95% CI: 0.197–0.236) than GY (0.182, 95% CI: 0.182–0.182) and QDN (0.155, 95% CI: 0.149–0.160). Bootstrap tests confirmed that LPS > GY (*H* difference 95% CI: 0.016–0.054, *p* = 0.0062) and LPS > QDN (0.042–0.082, *p* < 0.001). The raw comparison overestimated the LPS–GY difference (0.057 vs. 0.038 after rarefaction) but did not reverse the rank order. Among other populations, TR (*H*_rare = 0.206, 95% CI: 0.179–0.224) and QXN (0.196, 0.174–0.208) showed moderate diversity, whereas ZY (0.181, 0.161–0.193) and QN (0.193, 0.163–0.212) were similar to GY.

To better understand population genetic differentiation in *I. polycarpa*, we examined Nei’s genetic diversity. The results showed that the total genetic diversity (*Ht*) was 0.246 and the within-population diversity (*Hs*) was 0.215. This suggests a moderate level of overall genetic variation in this species, with most diversity found within populations. The coefficient of genetic differentiation (*Gst*) was 0.126, indicating that about 12.6% of the total genetic variation is partitioned among populations, reflecting low differentiation. The estimated gene flow (*Nm*) was 1.73, indicating moderate gene flow among populations. This level of gene flow may help counteract genetic drift and maintain genetic cohesion, although marker‑based estimates should be interpreted with caution (Table 2).

**Table 2:** *Nei’s* analysis of genetic diversity in *I. polycarpa* populations

*Ht*	*Hs*	*Gst*	*Nm*
0.246	0.215	0.126	1.73

*Ht,* total genetic diversity; *Hs*, within-population genetic diversity; *Gst*, genetic differentiation coefficient among populations; *Nm*, gene flow

The results of the Analysis of Molecular Variance (AMOVA) further support this pattern: variation among populations accounted for 11.20% of the total variance, whereas variation within populations accounted for 88.80%. Differentiation among populations was significant (*p* = 0.001). This suggests that a detectable, though modest, genetic structure has formed among *I. polycarpa* populations (Table 3).

**Table 3:** Analysis of molecular variance (AMOVA) among and within populations of *I. polycarpa*

Source	df	SS	Est. Var	Percentage of variation(%)	*p*-value
Among Pops	6	1253.8996	8.3347	11.1971	0.001
Within Pops	113	7469.5337	66.1021	88.8029	
Total	119	8723.4333	74.4368	100.0000	

In summary, genetic diversity levels vary across the different geographic populations of *I. polycarpa*. The LPS population appears to be a relatively high‑diversity hotspot, while the QDN population shows lower diversity. Additionally, the population genetic structure is characterized primarily by variation within populations, with low differentiation among populations.

### Genetic structure analysis of* I. polycarpa* in Guizhou Province

**Fig. 3:**
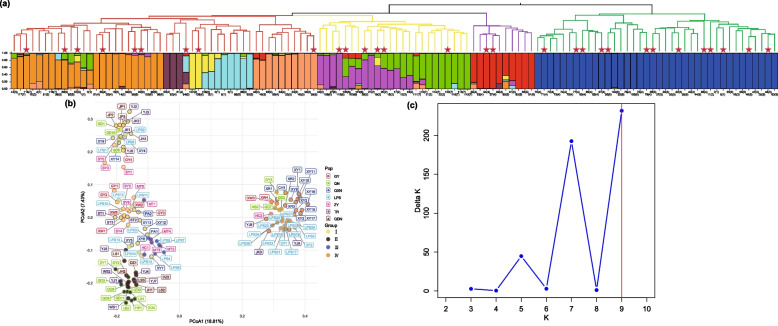
Genetic structure and clustering of *I. polycarpa* accessions in Guizhou Province. **a** Combined view of the UPGMA dendrogram (top) and population structure (bottom). In the UPGMA dendrogram, four major clusters (I–IV) are indicated by colored branch lines: red for cluster I, yellow for cluster II, purple for cluster III, and green for cluster IV. The bottom panel shows the population structure inferred by STRUCTURE at K = 9, with each individual represented by a vertical bar partitioned into nine colored segments proportional to its estimated ancestry coefficients. Red stars indicate the core collection. **b** Principal coordinate analysis (PCoA) of 120 accessions based on the Jaccard distance matrix. Each point represents an individual accession and is labeled with its sample ID. Colors correspond to the four UPGMA clusters: yellow for cluster I, black for cluster II, blue for cluster III, and orange for cluster IV. **c** Delta K (ΔK) values for assumed numbers of populations (K = 2–10) calculated using STRUCTURE v2.3.4, showing a peak at K = 9

To quantitatively assess genetic relationships, pairwise Jaccard similarity coefficients were calculated for all 120 accessions (Supplementary Table S7). The overall mean similarity was 0.243 ± 0.102 (range: 0.074–0.664). Within‑population mean similarities ranged from 0.256 (LPS) to 0.391 (QDN), whereas between‑population similarities were generally lower (Supplementary Table S8). Using the Jaccard distance matrix, UPGMA clustering was performed with combined SSR and IRAP markers (42 polymorphic primers). The dendrogram (Fig. 3a) resolved all accessions into four major clusters (I–IV) (Supplementary Table S9). Cluster I was the largest (48 accessions, 40% of the total) and included individuals from all seven geographic populations. Cluster IV (38 accessions) mainly comprised individuals from Qianxinan (QXN) and Liupanshui (LPS). Cluster II (24 accessions) was distributed across Qiannan (QN), Tongren (TR), and Qiandongnan (QDN). Cluster III (10 accessions) was the smallest and primarily originated from LPS and Zunyi (ZY). Principal coordinate analysis (PCoA) (Fig. 3b) corroborated the four groups observed in the UPGMA clustering.

Bayesian clustering in STRUCTURE was used to infer the number of ancestral populations. The ΔK statistic peaked at K = 9 (Fig. 3c), and the log probability L(K) plateaued at this value, indicating that the 120 accessions most likely originated from nine ancestral groups with distinct genetic backgrounds (Fig. 3a, Supplementary Table S10). With a membership coefficient threshold of Q ≥ 0.7, most individuals were assigned to a single ancestral group, whereas several admixed individuals (e.g., GY3, XW1, ST1, MT5, YJ5, GY1) showed Q values distributed across multiple groups, indicating complex genetic mixing.

Comparison of UPGMA clustering and STRUCTURE revealed a hierarchical genetic structure (Supplementary Table S10). UPGMA Cluster I (48 samples) primarily comprised individuals assigned to STRUCTURE Groups 1, 2, 3, 4, and 5, reflecting substantial ancestral diversity within this large cluster. UPGMA Cluster II (24 samples) aligned mainly with STRUCTURE Groups 6 and 7. UPGMA Cluster III (10 samples) closely matched STRUCTURE Group 8, with most individuals showing Q ≥ 0.8. UPGMA Cluster IV (38 samples) was strongly associated with STRUCTURE Group 9, with most individuals having Q > 0.99. Notably, Cluster I exhibited the greatest internal complexity, resolving into multiple distinct STRUCTURE groups, indicating that despite overall similarity, it harbors diverse ancestral backgrounds. In contrast, Clusters III and IV showed high consistency with single STRUCTURE groups, indicating more homogeneous genetic compositions. Samples from LPS were distributed across multiple UPGMA clusters and STRUCTURE groups, reflecting high genetic diversity and potentially serving as a contact zone for divergent lineages.

In summary, UPGMA clustering identified four major lineages based on overall genetic similarity, providing a higher‑order framework for genetic structure. STRUCTURE analysis further resolved these lineages into nine ancestral components, revealing fine‑scale genetic admixture and differentiation, particularly within the most heterogeneous cluster (Group I).

### Construction of the core collection for *I. polycarpa* in Guizhou Province

**Fig. 4:**
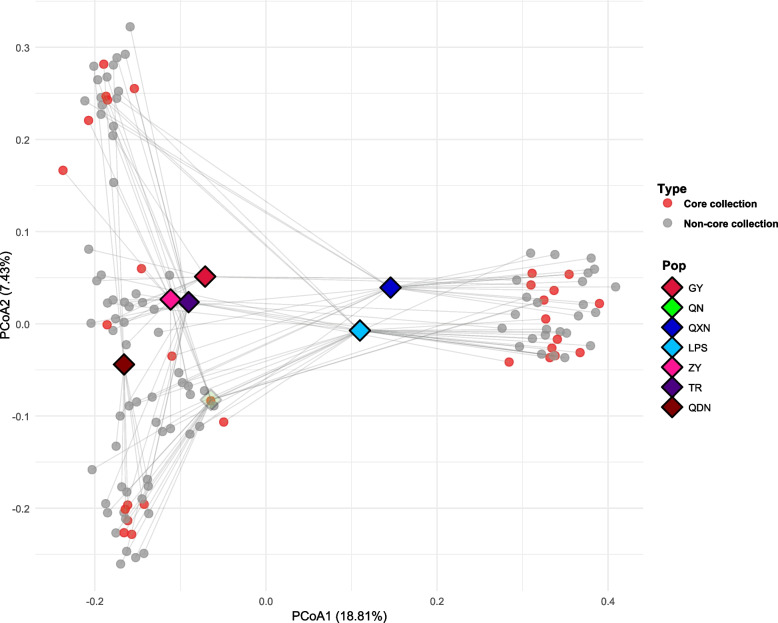
Principal coordinate analysis of core and original collections

Using PowerCore 1.0, we performed random sampling at 5%, 10%, 15%, 20%, 25%, and 30% of the total germplasm collection. To account for the unavoidable regional imbalance in the original sampling (sample sizes ranged from 9 to 29), we applied the allele-coverage-based M-strategy in PowerCore, which selects accessions to maximize distinct alleles regardless of population size or diversity indices, thereby minimizing bias toward larger populations. A one-way ANOVA was used to test for significant differences in genetic diversity parameters between each core subset and the original collection (Table 4). No significant differences were observed for subsets C5 (25%) and C6 (30%). To minimize redundancy and maximize representativeness, we recommend retaining the C5 subset, which consists of 30 *I. polycarpa* individuals (25% of the original 120 accessions). This core subset preserves 96.7% of the total alleles and, importantly, captures all private alleles (unique bands) from the smallest populations (GY and QDN). Principal Coordinate Analysis (PCoA) was used to assess the genetic diversity of both the core and the entire germplasm, confirming that the core germplasm broadly spans the geographic range of the whole collection (Fig. 4) and adequately represents the genetic makeup of the original germplasm population. Detailed information on the core germplasm is provided in Supplementary Table S11. The 30 core collections collectively represent all nine ancestral groups identified by STRUCTURE (Supplementary Table S12).

**Table 4:** Genetic diversity parameters of the constructed core collection and the original germplasm subsets as analyzed by one-way ANOVA

Populations	No	loci	*Na*	*Ne*	*H*	*I*
C0	120	596	2.00	1.39 ± 0.32	0.24 ± 0.16	0.56 ± 0.30
C1	6	406	2.00	1.62 ± 0.25^**^	0.37 ± 0.10^**^	0.79 ± 0.16^**^
C2	12	494	2.00	1.51 ± 0.29^**^	0.31 ± 0.13^**^	0.69 ± 0.22^**^
C3	18	533	2.00	1.47 ± 0.30^**^	0.29 ± 0.14^**^	0.65 ± 0.25^**^
C4	24	559	2.00	1.44 ± 0.31	0.27 ± 0.15^*^	0.62 ± 0.26^**^
C5	30	573	2.00	1.43 ± 0.31	0.27 ± 0.15	0.61 ± 0.27
C6	36	581	2.00	1.42 ± 0.31	0.26 ± 0.15	0.60 ± 0.28

C0 represents the original germplasm; C1-C6 are the core germplasm randomly selected in proportions of 5%, 10%, 15%, 20%, 25%, and 30%, respectively, based on the original germplasm

^*^Indicates a significant difference at the 0.05 level

^**^Indicates a highly significant difference at the 0.01 level

To enable fast germplasm identification and traceability, we also used the IRAP molecular marker system, which exhibits high overall polymorphism, to create unique DNA fingerprinting profiles for each core accession, represented as a binary (0/1) matrix. The results showed that primers RT19, RT23, RT24, RT27, and RT34 each achieved an identification rate of 93.33%. Using any two primers together provided 100% discrimination among all 30 core accessions. As a result, primers RT19 and RT34 were chosen to establish the DNA fingerprinting profiles for the core germplasm (Supplementary Table S13). For example, the fingerprint code for accession XW4 is “A00000000001001010110B000000000101000010000.” In this code, the letters “A” and “B” represent primers RT19 and RT34, respectively. The digits that follow each letter indicate the amplification bands of the primer, where “1” means the band is present and “0” means it is absent.

## Discussion

### Complementarity of SSR and IRAP markers in revealing genetic structure

In this study, 42 polymorphic primers (18 SSR and 24 IRAP) generated a total of 596 alleles with a 100% polymorphism rate across 120 *I. polycarpa* accessions. Consistent with previous reports [23, 26, 43–46], both marker types exhibited high discriminative power in evaluating genetic relationships. Notably, IRAP markers outperformed SSR markers across various genetic parameters, a superiority that may be due to the widespread distribution and high copy number of retrotransposons in the genome, which often offer richer polymorphism [46].

The unique sequence features of these two marker types offer an interesting opportunity to explore why they perform differently. In this study, SSR clustering effectively identified accessions with pubescent leaf undersides, whereas IRAP clustering showed no direct association with this trait. This difference aligns with findings in other species, in which SSR markers reveal evidence of selection associated with local adaptation [47, 48]. Conversely, IRAP markers reflect genome-wide retrotransposon insertion variations, providing a broader view of evolutionary history and structural genomic differences [49]. Therefore, for tree species like *I. polycarpa*, which have complex genetic backgrounds and potential adaptive differences, combining multiple marker types provides a more detailed, layered understanding of genetic structure. This approach helps avoid bias from relying on a single marker type, offering a more reliable foundation for germplasm assessment and conservation planning.

### Genetic diversity patterns and conservation implications

Genetic diversity was unevenly distributed among the seven *I. polycarpa* populations in Guizhou Province. The LPS population showed the highest values across all diversity measures, suggesting it could be a genetic diversity “hotspot” or a historical refuge for the species, and should therefore be a priority for conservation. Conversely, the QDN population exhibited the lowest diversity, indicating a potential risk of genetic erosion and requiring focused conservation efforts.

Genetic differentiation among populations was low (*Gst* = 0.126; 11.20% of variation among populations, as shown by AMOVA), while gene flow was moderate (*Nm* = 1.73). This pattern aligns with findings in many perennial woody plants [50–52] and likely reflects the biological traits of *I. polycarpa*, including its long lifespan, broad distribution, and seed-dispersal ability. Moderate gene flow likely helps maintain the species’ overall genetic unity. Conservation efforts should therefore focus on protecting high-diversity populations (e.g., LPS) and promoting gene flow between low- and high-diversity groups, as this strategy is essential for maintaining and enhancing the species’ overall adaptive genetic capacity.

### Hierarchical genetic structure revealed by multi-level analyses

In genetic diversity research, phylogenetic and population structure analyses together reveal genetic differentiation, evolutionary history, and gene flow patterns within populations or species [53–56]. In this study, combining UPGMA clustering, PCoA, and Bayesian STRUCTURE analysis uncovered a hierarchical genetic organization in *I. polycarpa* germplasm resources in Guizhou Province.

UPGMA clustering based on genetic distance and PCoA consistently divided the 120 accessions into four main clusters, creating a higher-level genetic framework with some correlation between geographical origin and genetic grouping. In contrast, STRUCTURE identified nine ancestral groups (K = 9) as the optimal partition, capturing more detailed genetic components. These four macro-clusters, nested within nine ancestral components, represent different levels of analysis rather than conflicting findings. The four UPGMA clusters correspond to major genetic lineages, defined by overall genetic similarity and geographic distribution, offering a clear framework for discussing primary divergence events. The nine STRUCTURE groups further detail sub-lineages or ancestral components within these lineages, providing insights into finer-scale admixture and local adaptation.

Notably, the largest UPGMA cluster (Group I) showed the highest internal diversity, comprising individuals from several ancestral backgrounds, whereas Groups III and IV appeared more uniform. This hierarchical arrangement may reflect the influence of gene flow (*Nm* = 1.73), local geographical separation, and historical migration events. Samples from the LPS region were spread across multiple clusters and subgroups in all analyses, aligning with this region having the greatest genetic diversity. This strongly indicates that LPS may be a key area for the mixing, retention, and divergence of genetic lineages, making it crucial for species evolution and conservation.

### Core collection development and DNA fingerprinting for effective conservation and identification of *I. polycarpa* germplasm resources

A core collection, representing a selected subset from a larger germplasm pool, plays a key role in conserving and utilizing wild plant resources [15, 57]. Using combined SSR and IRAP data, we constructed a core collection of 30 *I. polycarpa* accessions (25% of the original set) through PowerCore software, which employs the M-strategy to maximize allelic diversity while reducing genetic redundancy. This core subset retained the original genetic diversity with no significant loss of key diversity parameters, and PCoA confirmed its coverage of overall genetic and geographic variation [21, 57–59].

Additionally, highly polymorphic IRAP primers (RT19 and RT34) generated unique DNA fingerprint profiles for the core collection, achieving 100% individual discrimination. This fingerprinting system offers a molecular “ID card” for germplasm management, tracking, and exchange, and supports cultivar identification and intellectual property protection. In the future, this fingerprint database can be integrated with phenotypic trait databases to gradually build a comprehensive genotype–phenotype information system for *I. polycarpa*, enabling a shift from conservation toward efficient utilization.

### Limitations due to unbalanced sampling

Unbalanced sample sizes across regions (ranging from 9 to 29) were unavoidable given the fragmented distribution of *I. polycarpa*. Raw diversity indices correlated strongly with sample size (*r* = 0.909), but rarefaction eliminated this bias (*r* = 0.18). Despite this correction, the smallest populations (GY, QDN) may still have underestimated diversity because of their rarity. Importantly, the core collection was constructed using the allele-coverage-based M-strategy, which retained > 96% of total alleles and captured all private alleles from GY and QDN. Thus, while population-level comparisons should be interpreted with caution, the core collection remains robust. Future sampling should prioritize GY and QDN.

## Conclusion

This study performed a comprehensive genetic diversity analysis of 120 *I. polycarpa* germplasm samples collected from Guizhou Province using two molecular marker systems. It also initially established a core collection for Guizhou *I. polycarpa*. The research offers valuable information for breeders aiming to preserve and utilize the genetic diversity of *I. polycarpa*, supporting germplasm resource conservation, genetic research, and breeding efforts. Greater focus should be placed on the core collection to enhance the efficiency of germplasm exchange and utilization among *I. polycarpa* resources.

## Supplementary Information

Supplementary Material 1.

Supplementary Material 2.

## Data Availability

The raw datasets produced and examined during this study are included in Supplementary Table S14.
