# Supplementary material for: SSR and IRAP-based genetic diversity analysis for core collection of Idesia polycarpa
Source: BMC Plant Biol. 2026 May 28;26:1269. doi: 10.1186/s12870-026-09068-7 (PMC13403587; doi:10.1186/s12870-026-09068-7)
Supplement: Supplementary file 1 — Supplementary Material 1. [file 12870_2026_9068_MOESM1_ESM.zip › Supplementary Table S1.docx]

**Supplementary Table S1** Collection data form for wild *I. polycarpa* germplasm resources in Guizhou Province

| **No.** | **Source** | **Name of germplasm** | **Sample size** | **Longitude/E** | **Latitude/N** | **Altitude/m** | **Pop (To.)** |
| --- | --- | --- | --- | --- | --- | --- | --- |
| 1 | Guiyang City, Guizhou Province | GGY1~GGY4 | 4 | 106°43′~106°44′ | 26°29′~26°37′ | 1128~1249 | GY (9) |
| 2 | Xiuwen County, Guizhou Province | GXW1~GXW5 | 5 | 106°43′~106°44′ | 26°50′~26°51′ | 1288~1303 |  |
| 3 | Guiding County, Guizhou Province | GGD1~GGD11 | 11 | 107°11′~107°18′ | 26°17′~26°20′ | 918~1233 | QN (18) |
| 4 | Duyun City, Guizhou Province | GDY1~GDY3 | 3 | 107°19′~107°28′ | 26°13′~26°18′ | 885~1450 |  |
| 5 | Huishui County, Guizhou Province | GHS1~GHS2 | 2 | 106°45′~106°49′ | 26°04′~26°08′ | 1127~1324 |  |
| 6 | Libo County, Guizhou Province | GLB1~GLB2 | 2 | 107°48′~107°52′ | 25°29′~25°31′ | 827~944 |  |
| 7 | Xingyi City, Guizhou Province | GXY1~GXY17 | 17 | 104°47′~104°48′ | 24°56′~24°58′ | 1615~1717 | QXN (23) |
| 8 | Xingren City, Guizhou Province | GXR1~GXR3 | 3 | 105°06′~105°08′ | 25°20′~25°22′ | 1476~1722 |  |
| 9 | Puan County, Guizhou Province | GPA1~GPA2 | 2 | 104°57′~104°58′ | 25°25′~25°26′ | 1813~1837 |  |
| 10 | Ceheng County, Guizhou Province | GCH1 | 1 | 105°41′ | 24°58′ | 1148 |  |
| 11 | Liupanshui City, Guizhou Province | GLPS1~GLPS27 | 27 | 104°42′~104°55′ | 26°07′~26°35′ | 1584~2307 | LPS (29) |
| 12 | Dafang County, Guizhou Province | GDF1~GDF2 | 2 | 105°51′~106°00′ | 27°13′~27°14′ | 1444~1647 |  |
| 13 | Meitan County, Guizhou Province | GMT1~GMT5 | 5 | 107°32′~107°42′ | 27°52′~27°59′ | 826~965 | ZY (13) |
| 14 | Suiyang County, Guizhou Province | GSY1-6 | 6 | 106°58′~107°15′ | 28°01′~ | 28°15′ |  |
| 15 | Huichuan District, Guizhou Province | GHC1~GHC2 | 2 | 106°49′~106°50′ | 27°58′ | 1166~1275 |  |
| 16 | Jiangkou County, Guizhou Province | GJK1~GJK4 | 4 | 108°35′~108°54′ | 27°38′~27°50′ | 367~808 | TR (18) |
| 17 | Yinjiang County, Guizhou Province | GYJ1~GYJ9 | 9 | 108°23′~108°38′ | 27°38′~27°56′ | 683~1355 |  |
| 18 | Songtao County, Guizhou Province | GST1~GST3 | 3 | 109°08′~109°09′ | 28°19′~28°20′ | 736~764 |  |
| 19 | Wanshan District, Guizhou Province | GWS1~GWS2 | 2 | 109°18′~109°19′ | 27°32′ | 440~441 |  |
| 20 | Danzhai County, Guizhou Province | GDZ1~GDZ2 | 2 | 107°52′~107°54′ | 26°10′~26°13′ | 728~896 | QDN (10) |
| 21 | Jianhe County, Guizhou Province | GJH1~GJH2 | 2 | 108°25′ | 26°23′ | 892~930 |  |
| 22 | Jingpin County, Guizhou Province | GJP1~GJP3 | 3 | 109°01′~109°02′ | 26°34′~26°36′ | 655~744 |  |
| 23 | Leishan County, Guizhou Province | GLS1~GLS3 | 3 | 108°04′~108°12′ | 26°16′~26°18′ | 1041~1246 |  |
