# Supplementary material for: SSR and IRAP-based genetic diversity analysis for core collection of Idesia polycarpa
Source: BMC Plant Biol. 2026 May 28;26:1269. doi: 10.1186/s12870-026-09068-7 (PMC13403587; doi:10.1186/s12870-026-09068-7)
Supplement: Supplementary file 1 — Supplementary Material 1. [file 12870_2026_9068_MOESM1_ESM.zip › Supplementary Table S10.docx]

**Supplementary Table S10** Distribution and classification of 120 *I. polycarpa* individuals using Structure v.2.3.4

| Group (To.) | Samples | No. (Pop.) | Q-value distribution | | | | | | | | |
| --- | --- | --- | --- | --- | --- | --- | --- | --- | --- | --- | --- |
|  |  |  | 1 | 2 | 3 | 4 | 5 | 6 | 7 | 8 | 9 |
| Ⅰ (48) | XY14 | 41 (3) | **0.857** | 0.001 | 0 | 0.001 | 0.002 | 0 | 0.137 | 0.002 | 0.001 |
|  | JP3 | 117 (7) | **0.905** | 0.001 | 0.002 | 0.002 | 0.001 | 0 | 0.046 | 0.042 | 0.001 |
|  | JP1 | 115 (7) | **0.935** | 0 | 0.032 | 0.024 | 0 | 0 | 0.006 | 0.001 | 0 |
|  | GD1 | 10 (2) | **0.842** | 0.001 | 0 | 0.003 | 0.152 | 0 | 0 | 0.001 | 0 |
|  | GY4 | 4 (1) | **0.738** | 0.001 | 0.013 | 0.033 | 0.212 | 0 | 0.001 | 0.001 | 0 |
|  | XY4 | 31 (3) | **0.985** | 0 | 0.001 | 0.002 | 0.005 | 0.001 | 0.004 | 0.001 | 0.001 |
|  | JP2 | 116 (7) | **0.989** | 0.006 | 0.002 | 0 | 0 | 0.001 | 0.001 | 0.001 | 0 |
|  | XY9 | 36 (3) | **0.807** | 0 | 0.001 | 0.178 | 0.008 | 0.001 | 0.003 | 0.001 | 0 |
|  | SY5 | 89 (5) | **0.808** | 0 | 0.002 | 0.014 | 0 | 0 | 0.174 | 0.001 | 0 |
|  | SY1 | 85 (5) | **0.66** | 0.001 | 0.003 | 0.021 | 0.039 | 0.001 | 0.275 | 0.001 | 0 |
|  | SY3 | 87 (5) | **0.702** | 0.001 | 0.182 | 0.004 | 0.05 | 0.004 | 0.057 | 0.001 | 0 |
|  | GY1 | 1 (1) | **0.475** | 0.037 | 0.017 | 0.057 | 0.012 | 0 | 0.4 | 0.001 | 0 |
|  | GY3 | 3 (1) | **0.383** | 0 | 0.027 | 0.003 | 0.14 | 0 | 0.429 | 0.017 | 0 |
|  | LPS1 | 51 (4) | **0.991** | 0.003 | 0.002 | 0.001 | 0 | 0.001 | 0 | 0.001 | 0 |
|  | LPS8 | 58 (4) | **0.988** | 0.001 | 0.005 | 0.001 | 0 | 0.002 | 0.001 | 0.001 | 0 |
|  | LPS2 | 52 (4) | **0.985** | 0.005 | 0.001 | 0.004 | 0.001 | 0.002 | 0.001 | 0.002 | 0 |
|  | GD10 | 19 (2) | **0.979** | 0.002 | 0.001 | 0.014 | 0.003 | 0.001 | 0 | 0 | 0 |
|  | GD5 | 14 (2) | **0.914** | 0.072 | 0.009 | 0 | 0.001 | 0.001 | 0.001 | 0.002 | 0 |
|  | JK2 | 94 (6) | **0.959** | 0.001 | 0 | 0 | 0 | 0 | 0 | 0.038 | 0 |
|  | JK4 | 96 (6) | **0.927** | 0.004 | 0.001 | 0.001 | 0.001 | 0.063 | 0 | 0.001 | 0.002 |
|  | JK1 | 93 (6) | **0.955** | 0.001 | 0.005 | 0.001 | 0 | 0.036 | 0 | 0.001 | 0 |
|  | YJ6 | 102 (6) | **0.904** | 0.001 | 0 | 0 | 0 | 0.091 | 0.002 | 0.001 | 0 |
|  | YJ2 | 98 (6) | **0.993** | 0 | 0.004 | 0 | 0 | 0 | 0 | 0.001 | 0 |
|  | YJ3 | 99 (6) | **0.995** | 0.001 | 0 | 0.001 | 0 | 0.001 | 0.001 | 0.001 | 0 |
|  | LPS10 | 60 (4) | 0 | **0.998** | 0 | 0 | 0 | 0 | 0 | 0.001 | 0 |
|  | LPS12 | 62 (4) | 0.001 | **0.993** | 0.001 | 0.003 | 0.001 | 0.001 | 0 | 0.001 | 0 |
|  | LPS11 | 61 (4) | 0 | **0.998** | 0 | 0 | 0 | 0 | 0 | 0.001 | 0 |
|  | MT5 | 84 (5) | 0.004 | 0.326 | 0.001 | **0.454** | 0.143 | 0 | 0 | 0.071 | 0 |
|  | ST2 | 107 (6) | 0 | 0 | **0.997** | 0 | 0.001 | 0 | 0 | 0.001 | 0 |
|  | ST3 | 108 (6) | 0.001 | 0 | **0.978** | 0.015 | 0 | 0.001 | 0.003 | 0.001 | 0 |
|  | ST1 | 106 (6) | 0.001 | 0.002 | **0.481** | 0.455 | 0.002 | 0 | 0.057 | 0.001 | 0 |
|  | XW1 | 5 (1) | 0.001 | 0.001 | **0.509** | 0.486 | 0.001 | 0 | 0 | 0.001 | 0 |
|  | XW2 | 6 (1) | 0 | 0 | 0.148 | **0.839** | 0.003 | 0.002 | 0.006 | 0.001 | 0 |
|  | XW5 | 9 (1) | 0 | 0 | 0.001 | **0.958** | 0 | 0.001 | 0.038 | 0 | 0 |
|  | GY2 | 2 (1) | 0.001 | 0.001 | 0.001 | **0.994** | 0 | 0 | 0 | 0.002 | 0 |
|  | SY2 | 86 (5) | 0 | 0.001 | 0 | **0.996** | 0 | 0 | 0 | 0.001 | 0 |
|  | SY4 | 88 (5) | 0.001 | 0.001 | 0.056 | **0.848** | 0 | 0.002 | 0.091 | 0.001 | 0 |
|  | SY6 | 90 (5) | 0.002 | 0.022 | 0.019 | **0.954** | 0 | 0 | 0 | 0.001 | 0 |
|  | PA1 | 48 (3) | 0.003 | 0.001 | 0.001 | 0.001 | **0.841** | 0.003 | 0.007 | 0.141 | 0.001 |
|  | PA2 | 49 (3) | 0.006 | 0.001 | 0.004 | 0.002 | **0.805** | 0 | 0.001 | 0.181 | 0 |
|  | LPS13 | 63 (4) | 0 | 0 | 0.001 | 0.004 | **0.981** | 0.013 | 0 | 0.001 | 0 |
|  | LPS15 | 65 (4) | 0 | 0 | 0.002 | 0.002 | **0.935** | 0.017 | 0.041 | 0.001 | 0 |
|  | LPS14 | 64 (4) | 0 | 0.001 | 0.05 | 0.002 | **0.933** | 0.01 | 0.002 | 0.001 | 0 |
|  | XY5 | 32 (3) | 0.001 | 0.001 | 0.004 | 0 | **0.97** | 0 | 0.023 | 0 | 0 |
|  | XY6 | 33 (3) | 0.002 | 0.002 | 0.003 | 0.002 | **0.975** | 0.003 | 0.012 | 0.001 | 0 |
|  | XY13 | 40 (3) | 0.001 | 0.001 | 0.001 | 0.001 | **0.996** | 0 | 0 | 0.001 | 0 |
|  | XY7 | 34 (3) | 0 | 0.001 | 0 | 0 | **0.997** | 0 | 0 | 0.001 | 0 |
|  | XY12 | 39 (3) | 0 | 0.003 | 0 | 0 | **0.994** | 0 | 0 | 0.001 | 0 |
| Ⅱ (24) | HS1 | 24 (2) | 0 | 0 | 0.001 | 0.001 | 0.115 | **0.833** | 0.044 | 0.006 | 0 |
|  | WS1 | 109 (6) | 0 | 0 | 0.001 | 0.001 | 0 | **0.935** | 0.062 | 0.001 | 0 |
|  | YJ1 | 97 (6) | 0.003 | 0.051 | 0.036 | 0.005 | 0.007 | **0.895** | 0.002 | 0.002 | 0 |
|  | WS2 | 110 (6) | 0 | 0.001 | 0.001 | 0.001 | 0 | **0.996** | 0 | 0.001 | 0 |
|  | YJ7 | 103 (6) | 0.001 | 0 | 0.001 | 0.001 | 0 | **0.35** | 0.515 | 0.129 | 0.001 |
|  | YJ4 | 100 (6) | 0.004 | 0 | 0.003 | 0.017 | 0.001 | **0.618** | 0.219 | 0.136 | 0.001 |
|  | YJ5 | 101 (6) | 0.109 | 0.001 | 0.002 | 0.041 | 0 | **0.419** | 0.279 | 0.149 | 0 |
|  | LB1 | 26 (2) | 0.001 | 0.015 | 0.002 | 0.001 | 0.001 | **0.801** | 0.178 | 0.001 | 0 |
|  | LB2 | 27 (2) | 0 | 0 | 0 | 0 | 0 | **0.944** | 0.053 | 0.001 | 0 |
|  | GD9 | 18 (2) | 0.001 | 0 | 0.237 | 0 | 0.007 | **0.641** | 0.112 | 0.001 | 0 |
|  | GD11 | 20 (2) | 0.001 | 0.001 | 0.001 | 0.001 | 0 | **0.575** | 0.42 | 0.001 | 0 |
|  | GD6 | 15 (2) | 0 | 0.001 | 0.001 | 0 | 0.002 | **0.522** | 0.473 | 0.001 | 0 |
|  | GD8 | 17 (2) | 0.001 | 0.001 | 0.001 | 0.001 | 0 | **0.57** | 0.425 | 0.001 | 0 |
|  | GD3 | 12 (2) | 0.001 | 0 | 0 | 0 | 0 | 0.331 | **0.666** | 0 | 0 |
|  | GD4 | 13 (2) | 0 | 0 | 0.001 | 0 | 0.001 | 0.381 | **0.615** | 0.001 | 0 |
|  | DZ1 | 111 (7) | 0.003 | 0.001 | 0.007 | 0.005 | 0.163 | 0.035 | **0.712** | 0.073 | 0 |
|  | DZ2 | 112 (7) | 0.001 | 0 | 0.009 | 0.004 | 0.124 | 0.145 | **0.704** | 0.013 | 0 |
|  | DY1 | 21 (2) | 0.001 | 0.001 | 0.002 | 0.001 | 0.003 | 0 | **0.99** | 0.001 | 0 |
|  | JH2 | 114 (7) | 0 | 0 | 0.005 | 0 | 0 | 0.001 | **0.992** | 0.001 | 0 |
|  | DY2 | 22 (2) | 0.001 | 0 | 0 | 0.001 | 0.001 | 0.001 | **0.992** | 0.004 | 0 |
|  | LS3 | 120 (7) | 0 | 0 | 0.002 | 0 | 0.001 | 0.001 | **0.994** | 0.001 | 0 |
|  | LS1 | 118 (7) | 0.001 | 0 | 0 | 0.155 | 0.001 | 0.001 | **0.841** | 0.001 | 0 |
|  | JH1 | 113 (7) | 0 | 0 | 0.001 | 0.001 | 0.001 | 0.001 | **0.994** | 0.002 | 0 |
|  | LS2 | 119 (7) | 0 | 0 | 0 | 0 | 0.001 | 0.003 | **0.994** | 0.001 | 0 |
| Ⅲ (10) | LPS6 | 56 (4) | 0.002 | 0 | 0 | 0.001 | 0.136 | 0.046 | 0.001 | **0.813** | 0.001 |
|  | LPS3 | 53 (4) | 0 | 0.008 | 0 | 0 | 0 | 0 | 0 | **0.99** | 0 |
|  | LPS4 | 54 (4) | 0 | 0.01 | 0 | 0 | 0 | 0 | 0 | **0.988** | 0 |
|  | LPS7 | 57 (4) | 0 | 0.008 | 0.001 | 0.001 | 0 | 0 | 0 | **0.989** | 0 |
|  | LPS9 | 59 (4) | 0 | 0.156 | 0 | 0 | 0 | 0 | 0 | **0.842** | 0 |
|  | MT1 | 80 (5) | 0.207 | 0 | 0.126 | 0.043 | 0 | 0.001 | 0 | **0.622** | 0 |
|  | MT2 | 81 (5) | 0.001 | 0.002 | 0.007 | 0.097 | 0 | 0.131 | 0.001 | **0.76** | 0.001 |
|  | MT3 | 82 (5) | 0 | 0.001 | 0 | 0.001 | 0 | 0 | 0 | **0.997** | 0 |
|  | MT4 | 83 (5) | 0 | 0 | 0 | 0.001 | 0 | 0 | 0.001 | **0.997** | 0 |
|  | HC1 | 91 (5) | 0 | 0 | 0 | 0.001 | 0 | 0 | 0 | **0.997** | 0 |
| Ⅳ (38) | LPS20 | 70 (4) | 0 | 0 | 0 | 0 | 0 | 0 | 0 | 0.001 | **0.998** |
|  | LPS21 | 71 (4) | 0 | 0 | 0 | 0 | 0 | 0 | 0 | 0.001 | **0.998** |
|  | LPS22 | 72 (4) | 0 | 0 | 0 | 0 | 0 | 0 | 0 | 0.001 | **0.998** |
|  | LPS23 | 73 (4) | 0.001 | 0 | 0.001 | 0 | 0 | 0.001 | 0 | 0.001 | **0.996** |
|  | LPS24 | 74 (4) | 0 | 0 | 0 | 0 | 0 | 0 | 0 | 0.001 | **0.998** |
|  | LPS25 | 75 (4) | 0 | 0 | 0 | 0 | 0 | 0 | 0 | 0.001 | **0.997** |
|  | LPS27 | 77 (4) | 0 | 0.001 | 0 | 0 | 0 | 0 | 0 | 0.001 | **0.997** |
|  | LPS19 | 69 (4) | 0 | 0.001 | 0 | 0 | 0 | 0 | 0 | 0.002 | **0.996** |
|  | LPS18 | 68 (4) | 0 | 0 | 0 | 0 | 0 | 0 | 0 | 0.004 | **0.994** |
|  | LPS5 | 55 (4) | 0 | 0 | 0 | 0 | 0 | 0 | 0 | 0.001 | **0.998** |
|  | LPS17 | 67 (4) | 0 | 0 | 0 | 0 | 0 | 0 | 0 | 0.001 | **0.998** |
|  | JK3 | 95 (6) | 0.001 | 0.001 | 0 | 0.001 | 0 | 0.002 | 0.006 | 0.002 | **0.985** |
|  | YJ8 | 104 (6) | 0.002 | 0 | 0.028 | 0.007 | 0 | 0.001 | 0.006 | 0.002 | **0.952** |
|  | HC2 | 92 (5) | 0.001 | 0.001 | 0.001 | 0.001 | 0 | 0.001 | 0.001 | 0.001 | **0.992** |
|  | LPS26 | 76 (4) | 0 | 0 | 0.001 | 0 | 0.001 | 0.001 | 0.001 | 0.001 | **0.995** |
|  | YJ9 | 105 (6) | 0 | 0 | 0 | 0 | 0 | 0 | 0 | 0 | **0.998** |
|  | DF1 | 78 (4) | 0 | 0 | 0 | 0 | 0 | 0 | 0 | 0 | **0.997** |
|  | DF2 | 79 (4) | 0 | 0 | 0 | 0 | 0 | 0 | 0.001 | 0 | **0.998** |
|  | XY8 | 35 (3) | 0 | 0 | 0 | 0 | 0 | 0 | 0 | 0 | **0.998** |
|  | XY10 | 37 (3) | 0 | 0 | 0 | 0 | 0 | 0 | 0 | 0 | **0.998** |
|  | XY2 | 29 (3) | 0 | 0 | 0 | 0 | 0 | 0 | 0 | 0 | **0.998** |
|  | XY3 | 30 (3) | 0 | 0 | 0 | 0 | 0 | 0 | 0 | 0.001 | **0.998** |
|  | XY15 | 42 (3) | 0 | 0 | 0 | 0 | 0 | 0 | 0 | 0 | **0.998** |
|  | XY11 | 38 (3) | 0 | 0 | 0 | 0 | 0 | 0 | 0 | 0 | **0.998** |
|  | XY16 | 43 (3) | 0 | 0 | 0 | 0 | 0 | 0 | 0 | 0 | **0.998** |
|  | XY17 | 44 (3) | 0 | 0 | 0 | 0 | 0 | 0 | 0 | 0.001 | **0.998** |
|  | LPS16 | 66 (4) | 0 | 0 | 0 | 0 | 0 | 0 | 0 | 0 | **0.997** |
|  | GD2 | 11 (2) | 0 | 0 | 0 | 0 | 0.001 | 0 | 0.002 | 0 | **0.995** |
|  | XW3 | 7 (1) | 0.003 | 0 | 0.001 | 0.002 | 0.001 | 0.001 | 0.001 | 0.001 | **0.992** |
|  | XW4 | 8 (1) | 0.006 | 0.001 | 0.003 | 0.002 | 0 | 0.001 | 0 | 0.002 | **0.985** |
|  | DY3 | 23 (2) | 0.001 | 0 | 0 | 0 | 0 | 0 | 0 | 0.001 | **0.996** |
|  | GD7 | 16 (2) | 0 | 0 | 0.001 | 0.001 | 0.001 | 0 | 0.001 | 0.001 | **0.996** |
|  | HS2 | 25 (2) | 0 | 0 | 0 | 0.001 | 0.001 | 0 | 0.001 | 0.001 | **0.996** |
|  | XR2 | 46 (3) | 0 | 0 | 0 | 0 | 0 | 0 | 0 | 0 | **0.997** |
|  | XR3 | 47 (3) | 0 | 0 | 0 | 0 | 0.001 | 0 | 0 | 0 | **0.998** |
|  | XY1 | 28 (3) | 0 | 0 | 0 | 0 | 0 | 0 | 0 | 0 | **0.998** |
|  | XR1 | 45 (3) | 0 | 0 | 0 | 0.001 | 0.001 | 0 | 0 | 0.001 | **0.996** |
|  | CH1 | 50 (3) | 0 | 0 | 0 | 0 | 0.001 | 0 | 0 | 0 | **0.998** |

Group (To.) indicates the division of samples into four major groups (Group I-IV) based on UPGMA clustering, along with the number of samples in each group. No. (Pop.) represents the sample codes and their corresponding collection locations. The Q-value distribution shows the membership probability of each sample to the nine ancestral clusters in the STRUCTURE analysis (K=9). The Q-value in bold indicates the optimal ancestral cluster for that sample.
