# Supplementary material for: SSR and IRAP-based genetic diversity analysis for core collection of Idesia polycarpa
Source: BMC Plant Biol. 2026 May 28;26:1269. doi: 10.1186/s12870-026-09068-7 (PMC13403587; doi:10.1186/s12870-026-09068-7)
Supplement: Supplementary file 1 — Supplementary Material 1. [file 12870_2026_9068_MOESM1_ESM.zip › Supplementary Table S11.docx]

**Supplementary Table S11** 30 core germplasm resources of *I. polycarpa*

| Number | Sample ID | Source | Location |
| --- | --- | --- | --- |
| 1 | DF2 | Dafang, Guizhou | 27°13′58″N 105°51′56″E |
| 2 | GD2 | Guiding, Guizhou | 26°17′38″N 107°11′51″E |
| 3 | GD9 |  | 26°17′20″N 107°12′16″E |
| 4 | GD11 |  | 26°18′54″N 107°16′40″E |
| 5 | JK1 | Jiangkou, Guizhou | 27°44′14″N 108°54′50″E |
| 6 | JK3 |  | 27°50′29″N 108°46′29″E |
| 7 | JK4 |  | 27°38′32″N 108°35′02″E |
| 8 | JP1 | Jinping, Guizhou | 26°34′33″N 109°01′44″E |
| 9 | LB1 | Libo, Guizhou | 25°29′21″N 107°48′36″E |
| 10 | LPS4 | Liupanshui, Guizhou | 26°23′15″N 104°51′53″E |
| 11 | LPS7 |  | 26°22′26″N 104°51′54″E |
| 12 | LPS8 |  | 26°22′18″N 104°51′53″E |
| 13 | LPS16 |  | 26°19′40″N 104°55′15″E |
| 14 | LPS17 |  | 26°21′46″N 104°52′36″E |
| 15 | LPS19 |  | 26°21′51″N 104°52′31″E |
| 16 | LPS21 |  | 26°22′29″N 104°53′01″E |
| 17 | LPS27 |  | 26°35′31″N 104°45′43″E |
| 18 | LS3 | Leishan, Guizhou | 26°18′11″N 108°12′34″E |
| 19 | MT5 | Meitang, Guizhou | 27°58′14″N 107°33′27″E |
| 20 | ST3 | Songtao, Guizhou | 28°19′33″N 109°08′59″E |
| 21 | SY3 | Suiyang, Guizhou | 28°06′56″N 106°58′39″E |
| 22 | SY5 |  | 28°12′60″N 106°59′55″E |
| 23 | WS2 | Wanshan, Guizhou | 27°32′02″N 109°18′10″E |
| 24 | XR1 | Xingren, Guizhou | 25°22′19″N 105°06′52″E |
| 25 | XR2 |  | 25°22′44″N 105°07′47″E |
| 26 | XW4 | Xiuwen, Guizhou | 26°50′29″N 106°43′07″E |
| 27 | XY8 | Xingyi, Guizhou | 24°56′45″N 104°47′56″E |
| 28 | XY12 |  | 24°56′30″N 104°47′04″E |
| 29 | XY15 |  | 24°56′25″N 104°47′32″E |
| 30 | YJ7 | Yinjiang, Guizhou | 27°41′59″N 108°31′05″E |
