# Supplementary material for: SSR and IRAP-based genetic diversity analysis for core collection of Idesia polycarpa
Source: BMC Plant Biol. 2026 May 28;26:1269. doi: 10.1186/s12870-026-09068-7 (PMC13403587; doi:10.1186/s12870-026-09068-7)
Supplement: Supplementary file 1 — Supplementary Material 1. [file 12870_2026_9068_MOESM1_ESM.zip › Supplementary Table S12.docx]

**Supplementary Table S12** Coverage of the nine ancestral groups in the core collection

| Core collection | Ancestral group (majority Q) | Q value | Remarks |
| --- | --- | --- | --- |
| LPS8 | Group 1 | 0.988 | Pure |
| LPS4 | Group 8 | 0.988 | Pure |
| LPS7 | Group 8 | 0.989 | Pure |
| LPS16 | Group 9 | 0.997 | Pure |
| LPS17 | Group 9 | 0.998 | Pure |
| LPS19 | Group 9 | 0.996 | Pure |
| LPS21 | Group 9 | 0.998 | Pure |
| LPS27 | Group 9 | 0.997 | Pure |
| DF2 | Group 9 | 0.997 | Pure |
| GD2 | Group 9 | 0.995 | Pure |
| GD9 | Mixed (Group 6: 0.641, Group 3: 0.237, Group 7: 0.112) | <0.7 | Contains Groups 3, 6, 7 |
| GD11 | Mixed (Group 6: 0.575, Group 7: 0.420) | <0.7 | Contains Group 6 & 7 |
| JK1 | Group 1 | 0.955 | Pure |
| JK3 | Group 9 | 0.985 | Pure |
| JK4 | Group 1 | 0.927 | Pure |
| JP1 | Group 1 | 0.935 | Pure |
| LB1 | Group 6 | 0.801 | Pure |
| LS3 | Group 7 | 0.994 | Pure |
| MT5 | Mixed (Group 4: 0.454, Group 2: 0.326) | <0.7 | Contains Group 2 & 4 |
| ST3 | Group 3 | 0.978 | Pure |
| SY3 | Group 1 | 0.702 | Pure |
| SY5 | Group 1 | 0.808 | Pure |
| WS2 | Group 6 | 0.996 | Pure |
| XR1 | Group 9 | 0.996 | Pure |
| XR2 | Group 9 | 0.997 | Pure |
| XW4 | Group 9 | 0.985 | Pure |
| XY8 | Group 9 | 0.998 | Pure |
| XY12 | Group 5 | 0.994 | Pure |
| XY15 | Group 9 | 0.998 | Pure |
| YJ7 | Mixed (Group 7: 0.515, Group 6: 0.35, Group 8: 0.129) | <0.7 | Contains Groups 6,7,8 |

In the table above, “pure” refers to maximum Q ≥ 0.7; “admixed” refers to maximum Q < 0.7. Each ancestral group has at least one individual carrying its major component (either pure or admixed). Ancestral groups 2, 3, 4, and 5 are covered by individuals such as MT5, ST3, XY12, etc.; ancestral group 7 is purely covered by LS3.
