# Supplementary material for: SSR and IRAP-based genetic diversity analysis for core collection of Idesia polycarpa
Source: BMC Plant Biol. 2026 May 28;26:1269. doi: 10.1186/s12870-026-09068-7 (PMC13403587; doi:10.1186/s12870-026-09068-7)
Supplement: Supplementary file 1 — Supplementary Material 1. [file 12870_2026_9068_MOESM1_ESM.zip › Supplementary Table S13.docx]

**Supplementary Table S13** DNA fingerprinting codes of 30 copies of the core collection

| Number | Sample ID | DNA fingerprint code |
| --- | --- | --- |
| 1 | XW4 | A00000000001001010110B000000000101000010000 |
| 2 | GD2 | A00000010101000010000B000100000000010010010 |
| 3 | GD9 | A00000000010010010010B010010000010010011010 |
| 4 | GD11 | A00001000000010010110B000001000010100011100 |
| 5 | LB1 | A00100000000000010110B010001000010001101100 |
| 6 | XY8 | A00000000101011000010B000001000100010010000 |
| 7 | XY12 | A10000000000010100010B100010000100011101100 |
| 8 | XY15 | A00000000001001000010B000000000100010100000 |
| 9 | XR1 | A00000000101001000000B000000100100010110000 |
| 10 | XR2 | A00000000100011000010B000000000100010010000 |
| 11 | LPS4 | A00001000010001010111B000010001000000001000 |
| 12 | LPS7 | A00000100000101001110B100010001000000001000 |
| 13 | LPS8 | A00000000000101100010B010011001100110010100 |
| 14 | LPS16 | A01000000001001000010B000000110000010010000 |
| 15 | LPS17 | A00010100000011000110B000001000001000010010 |
| 16 | LPS19 | A00010010000011000110B000101000000010010110 |
| 17 | LPS21 | A00000100000000010110B000001000001000010010 |
| 18 | LPS27 | A00000100001001000110B000100010001000010010 |
| 19 | DF2 | A00000010000011010010B000000100001000010000 |
| 20 | MT5 | A10000000000011100010B010010001000100110000 |
| 21 | SY3 | A00000000000101100001B001000001000100101100 |
| 22 | SY5 | A00000000000011011010B000010001000100110001 |
| 23 | JK1 | A00000000000101101010B010010001000100110110 |
| 24 | JK3 | A00010000000010010010B000001100001000010010 |
| 25 | JK4 | A00000001000101100010B000001001010100101100 |
| 26 | YJ7 | A00000000000000001011B010001000010010011100 |
| 27 | ST3 | A00000000000010101111B010010000000100001000 |
| 28 | WS2 | A00100000000100010110B000011000010001101100 |
| 29 | JP1 | A00000000000101101010B000010001000000001100 |
| 30 | LS3 | A00000001000011011010B110001000010001011100 |
