# Supplementary material for: SSR and IRAP-based genetic diversity analysis for core collection of Idesia polycarpa
Source: BMC Plant Biol. 2026 May 28;26:1269. doi: 10.1186/s12870-026-09068-7 (PMC13403587; doi:10.1186/s12870-026-09068-7)
Supplement: Supplementary file 1 — Supplementary Material 1. [file 12870_2026_9068_MOESM1_ESM.zip › Supplementary Table S2.docx]

**Supplementary Table S2** Information on 24 IRAP and 18 pairs of SSR primers for *I. polycarpa*

| No. | Primer name (IRAP) | Primer Sequence (5'→3') | Tm / ℃ | Primer name (SSR) | Repeat Motif | Primer Sequence (5'→3') |
| --- | --- | --- | --- | --- | --- | --- |
| 1 | RT4 | GAAGAATGCGTTCTTGCATGGG | 40.6 | SSR2 | (GA)6 | F: TTCTGCGATTCTACGGCTGC  R: CCTCTCGCTCGCTAAAACCC |
| 2 | RT6 | GTCGAATGCGTTTCTACATGGA | 43.9 | SSR4 | (AT)6 | F: AGGTGACATCCATCCTCCAGG  R: CGACCTCATTCTAGAGATTGTTGGG |
| 3 | RT7 | AGACTGCTTTTCTTCATGGA | 40 | SSR9 | (TTCT)6 | F: TCAAGATTTGCTCCGTATTTCACC  R: TCCCCTCACTATATGTTCTTGCC |
| 4 | RT8 | GCACAATGCTTTTCTCCATGGT | 40 | SSR17 | (AGAA)5 | F: CCAGTTCCTACAGCAAGGATCC  R: GCATCCATTCTCAAGGTTTCACG |
| 5 | RT9 | AGTCAACCTTCTTACATGGT | 40 | SSR19 | (TTTC)7 | F: TCCCAGGTGAATAGTTGAATTCC  R: CACGACTCAAGAGTTATATCAATGC |
| 6 | RT10 | GTCGAATGCATTTCTACATGGA | 40 | SSR20 | (CTT)5 | F: TCACCAAGAACCTATAGATACACC  R: TCTTCCTTAGTGAACTCGATGG |
| 7 | RT11 | AAAGACGGCTTTTCTTCATGGA | 40 | SSR21 | (ATAA)5 | F: AGGATTCCGAGGAAACATGTGC  R: GTGGGGTAGTTTCCTTGCCG |
| 8 | RT12 | CAAAACTGCATTTCTGAATGGG | 40 | SSR24 | (GGT)5 | F: TTAGGAGGTCGAGTTGGGGC  R: GCCGTTGCTGTTTTCTACATGC |
| 9 | RT15 | GAAGAGTGCATTTCTGCATAGT | 40 | SSR26 | (GAC)5 | F: GAAAACGGCTGTTGATCGCG  R: TCCCTCTCCTCCTTAGAAACTTCC |
| 10 | RT18 | AGCTCTCTATGGTCTAAAGCAA | 40 | SSR31 | (ATAC)6 | F: ACTTTTAGTTGAAAATGGAGGGAGG  R: AGTTCGTTCATGATCATTAGACACC |
| 11 | RT19 | AGCCTTGTATGGATTGAAGCAA | 43.9 | SSR35 | (CCA)6 | F: ACAACACAATCAGTAACCAGTTTCC  R: TTGCCACAATTTCCAGCTGC |
| 12 | RT21 | CCATATATGGCTTGAAACAG | 43.9 | SSR36 | (GCA)7 | F: GGTAACGTTGATTTGAAGTCCTTGG  R: CAGTGACAAAACCGCTTCTGC |
| 13 | RT23 | GGCTCTTTACGGCTTAAAGCAA | 40 | SSR38 | (CAAA)5 | F: GGCAACTATTACCCTTCTTTTGGC  R: CACGGATTTCTTAGAAACTTGGTGG |
| 14 | RT24 | AGCCCTCTACGGCTTGAAGCAA | 40 | SSR41 | (GTG)9 | F: GCTTCTGATAGCTCTTGTCTTGAGG  R: CGCAAATGGCCAGAATCATGC |
| 15 | RT26 | GGCACTTTATGGCTTAAAACAG | 40 | SSR42 | (TCA)8 | F: CCTAAGTGCACGCGACTAGG  R: CATGGCAACTGCAACTATGGC |
| 16 | RT27 | CACTATATGGCCTTAAACAG | 40 | SSR49 | (GCT)6 | F: GTACGGTGGAGTGTGGAAGG  R: CTTCAATCATGACTCAAGACGTCG |
| 17 | RT29 | AGCCATCTATGGTCTCAAGCAA | 40 | SSR52 | (CAGCAC)5 | F: CCAAAGACCAAATAGGCAAATGGG  R: TGTATCGTTTGGGAGTTGGTGG |
| 18 | RT30 | GGCCCTATACGGACTCAAACAA | 43.9 | SSR55 | (TCCC)5 | F: ACATCAATCACCATTATTGAACCCG  R: TGGTGGGATCCATTACCATTACC |
| 19 | RT31 | CTCTATATGGTCTCAAACAG | 40 |  |  |  |
| 20 | RT33 | ATCACTTTACGGACTCAAGCAA | 40 |  |  |  |
| 21 | RT34 | AGCCTTGTATGGCCTGAAGCAA | 40 |  |  |  |
| 22 | RT37 | TGTACGTGGATGACATATTG | 40 |  |  |  |
| 23 | RT41 | AGATGACATCCTGGTCTACTCA | 40 |  |  |  |
| 24 | RT60 | GCTAAGCTAAGCAAGTGTGAGT | 40.6 |  |  |  |
