# Supplementary material for: SSR and IRAP-based genetic diversity analysis for core collection of Idesia polycarpa
Source: BMC Plant Biol. 2026 May 28;26:1269. doi: 10.1186/s12870-026-09068-7 (PMC13403587; doi:10.1186/s12870-026-09068-7)
Supplement: Supplementary file 1 — Supplementary Material 1. [file 12870_2026_9068_MOESM1_ESM.zip › Supplementary Table S3.docx]

**Supplementary Table S3** Amplification, separation, and band scoring conditions for SSR and IRAP markers

| **Parameter** | **SSR Markers** | **IRAP Markers** |
| --- | --- | --- |
| **PCR Reaction Volume** | 10 μL total | 10 μL total |
| **Reaction Components** | - 5 μL mix - 0.5 μL each primer (10 μmol/L) - 1 μL template (15–20 ng/μL) - ddH₂O to volume | - 5 μL mix - **1 μL each primer** (10 μmol/L) - 1 μL template (15–20 ng/μL) - ddH₂O to volume |
| **PCR Strategy** | Touch-down PCR | **Annealing temperature gradient optimization** per primer |
| **Amplification Program** | 1. **Pre-denaturation**: 94°C, 4 min 2. **Touch-down phase (20 cycles):** - Denaturation: 94°C, 30 sec - Annealing: **65°C → 55°C** (↓0.5°C/cycle) - Extension: 72°C, 45 sec 3. **Standard phase (30–35 cycles):** - Denaturation: 94°C, 30 sec - Annealing: **55°C**, 30 sec - Extension: 72°C, 45 sec 4. **Final extension**: 72°C, 5 min 5. Hold at 4°C | 1. **Pre-denaturation**: 94°C, 4 min 2. **35 cycles:** - Denaturation: 94°C, 30 sec - Annealing: **Primer-specific Tm**, 45 sec - Extension: 72°C, **1 min** 3. **Final extension**: 72°C, **7 min** 4. Hold at 4°C |
| Separation conditions | 1. **2.0% agarose gel electrophoresis**  - 1× TAE buffer  - 120V 40min 2. **Qsep100 Automated Analysis** | **1.5% agarose gel electrophoresis**  - 1× TAE buffer  - 120V 40min |
| **Band scoring** | Band acquisition using the Qsep 100 Automated Nucleic Acid and Protein Analyzer | **Band scoring of electrophoresis results was performed using Gel-Pro 4.0 software with manual assistance** |

Note: Both systems use the same master mix (Tiangen Biotech (Beijing) Co., Ltd.) and template DNA concentration.
