# Supplementary material for: SSR and IRAP-based genetic diversity analysis for core collection of Idesia polycarpa
Source: BMC Plant Biol. 2026 May 28;26:1269. doi: 10.1186/s12870-026-09068-7 (PMC13403587; doi:10.1186/s12870-026-09068-7)
Supplement: Supplementary file 1 — Supplementary Material 1. [file 12870_2026_9068_MOESM1_ESM.zip › Supplementary Table S4.docx]

**Supplementary Table S4** Amplification results and polymorphism of SSR and IRAP primers

|  | Loci | PPL | *Na* | *Ne* | *H* | *I* | PIC |
| --- | --- | --- | --- | --- | --- | --- | --- |
| SSR |  |  |  |  |  |  |  |
| SSR2 | 18 | 100 | 2.00 | 1.19 | 0.14 | 0.37 | 0.90 |
| SSR4 | 9 | 100 | 2.00 | 1.26 | 0.16 | 0.38 | 0.65 |
| SSR9 | 6 | 100 | 2.00 | 1.30 | 0.20 | 0.46 | 0.54 |
| SSR17 | 8 | 100 | 2.00 | 1.31 | 0.19 | 0.43 | 0.66 |
| SSR19 | 7 | 100 | 2.00 | 1.41 | 0.25 | 0.57 | 0.70 |
| SSR20 | 5 | 100 | 2.00 | 1.47 | 0.29 | 0.65 | 0.65 |
| SSR24 | 10 | 100 | 2.00 | 1.21 | 0.13 | 0.33 | 0.63 |
| SSR26 | 8 | 100 | 2.00 | 1.32 | 0.20 | 0.46 | 0.72 |
| SSR31 | 16 | 100 | 2.00 | 1.20 | 0.14 | 0.35 | 0.84 |
| SSR35 | 7 | 100 | 2.00 | 1.35 | 0.21 | 0.49 | 0.65 |
| SSR36 | 16 | 100 | 2.00 | 1.21 | 0.16 | 0.39 | 0.88 |
| SSR38 | 5 | 100 | 2.00 | 1.53 | 0.33 | 0.72 | 0.67 |
| SSR41 | 12 | 100 | 2.00 | 1.24 | 0.16 | 0.38 | 0.77 |
| SSR42 | 13 | 100 | 2.00 | 1.27 | 0.18 | 0.45 | 0.85 |
| SSR45 | 7 | 100 | 2.00 | 1.31 | 0.19 | 0.44 | 0.61 |
| SSR49 | 10 | 100 | 2.00 | 1.31 | 0.19 | 0.44 | 0.70 |
| SSR52 | 8 | 100 | 2.00 | 1.44 | 0.27 | 0.60 | 0.77 |
| SSR55 | 7 | 100 | 2.00 | 1.32 | 0.18 | 0.42 | 0.57 |
| Total | 172 | - | - | - | - | - | - |
| Mean | 9.56 | 100 | 2.00 | 1.31 | 0.20 | 0.46 | 0.71 |
| IRAP |  |  |  |  |  |  |  |
| RT4 | 18 | 100 | 2.00 | 1.45 | 0.28 | 0.62 | 0.90 |
| RT6 | 18 | 100 | 2.00 | 1.46 | 0.28 | 0.63 | 0.90 |
| RT7 | 14 | 100 | 2.00 | 1.54 | 0.32 | 0.70 | 0.88 |
| RT8 | 17 | 100 | 2.00 | 1.42 | 0.26 | 0.59 | 0.88 |
| RT9 | 20 | 100 | 2.00 | 1.34 | 0.22 | 0.52 | 0.90 |
| RT10 | 17 | 100 | 2.00 | 1.44 | 0.26 | 0.59 | 0.89 |
| RT11 | 15 | 100 | 2.00 | 1.30 | 0.21 | 0.50 | 0.82 |
| RT12 | 13 | 100 | 2.00 | 1.41 | 0.24 | 0.54 | 0.83 |
| RT15 | 17 | 100 | 2.00 | 1.42 | 0.27 | 0.61 | 0.89 |
| RT18 | 18 | 100 | 2.00 | 1.44 | 0.28 | 0.64 | 0.90 |
| RT19 | 20 | 100 | 2.00 | 1.38 | 0.24 | 0.55 | 0.89 |
| RT21 | 16 | 100 | 2.00 | 1.50 | 0.30 | 0.66 | 0.90 |
| RT23 | 19 | 100 | 2.00 | 1.43 | 0.27 | 0.61 | 0.90 |
| RT24 | 20 | 100 | 2.00 | 1.49 | 0.29 | 0.64 | 0.91 |
| RT26 | 17 | 100 | 2.00 | 1.32 | 0.22 | 0.52 | 0.86 |
| RT27 | 18 | 100 | 2.00 | 1.46 | 0.28 | 0.62 | 0.89 |
| RT29 | 15 | 100 | 2.00 | 1.52 | 0.31 | 0.69 | 0.89 |
| RT30 | 19 | 100 | 2.00 | 1.50 | 0.31 | 0.68 | 0.91 |
| RT31 | 20 | 100 | 2.00 | 1.37 | 0.24 | 0.55 | 0.89 |
| RT33 | 16 | 100 | 2.00 | 1.57 | 0.34 | 0.74 | 0.90 |
| RT34 | 21 | 100 | 2.00 | 1.55 | 0.33 | 0.72 | 0.93 |
| RT37 | 18 | 100 | 2.00 | 1.38 | 0.24 | 0.55 | 0.89 |
| RT41 | 20 | 100 | 2.00 | 1.33 | 0.21 | 0.50 | 0.90 |
| RT60 | 18 | 100 | 2.00 | 1.43 | 0.26 | 0.59 | 0.89 |
| Total | 424 | - | - | - | - | - | - |
| Mean | 17.67 | 100 | 2.00 | 1.43 | 0.27 | 0.61 | 0.89 |

Loci, Loci number; PPL, Percentage of polymorphic Loci; *Na*, Observed number of alleles; *Ne*, effective allele number; *H*, Nei’s gene diversity; *I*, Shannon’s Information index; PIC, Polymorphism information content.
