# Supplementary material for: SSR and IRAP-based genetic diversity analysis for core collection of Idesia polycarpa
Source: BMC Plant Biol. 2026 May 28;26:1269. doi: 10.1186/s12870-026-09068-7 (PMC13403587; doi:10.1186/s12870-026-09068-7)
Supplement: Supplementary file 1 — Supplementary Material 1. [file 12870_2026_9068_MOESM1_ESM.zip › Supplementary Table S5.docx]

**Supplementary Table S5** Clustering classification of 120 *I. polycarpa* germplasm resources based on 18 SSR markers

| Group | To. | GY | QN | QXN | LPS | ZY | TR | QDN |
| --- | --- | --- | --- | --- | --- | --- | --- | --- |
| Ⅰ | 21 |  |  |  | LPS1, LPS2, LPS3, LPS4, LPS5, LPS7, LPS8, LPS9, LPS10, LPS11, LPS12, LPS17, LPS18, LPS19, LPS20, LPS21, LPS22, LPS23, LPS24, LPS25, LPS27 |  |  |  |
| Ⅱ | 2 | GY3, XW4 |  |  |  |  |  |  |
| Ⅲ | 3 |  |  |  |  | HC2 | JK3, YJ8 |  |
| Ⅳ | 20 | GY1, GY4 | DY3, GD5, GD10 | XY4, XY9, XY14 |  | SY1, SY3, SY5 | JK1, JK2, JK4, YJ2, YJ3, YJ6 | JP1, JP2, JP3 |
| Ⅴ | 74 | GY2, XW1, XW2, XW3, XW5 | DY1, DY2, GD1, GD2, GD3, GD4, GD6, GD7, GD8, GD9, GD11, HS1, HS2, LB1, LB2 | CH1, PA1, PA2, XR1, XR2, XR3, XY1, XY2, XY3, XY5, XY6, XY7, XY8, XY10, XY11, XY12, XY13, XY15, XY16, XY17 | DF1, DF2, LPS6, LPS13, LPS14, LPS15, LPS16, LPS26 | HC1, MT1, MT2, MT3, MT4, MT5, SY2, SY4, SY6 | ST1, ST2, ST3, WS1, WS2, YJ1, YJ4, YJ5, YJ7, YJ9 | DZ1, DZ2, JH1, JH2, LS1, LS2, LS3 |
