# Supplementary material for: SSR and IRAP-based genetic diversity analysis for core collection of Idesia polycarpa
Source: BMC Plant Biol. 2026 May 28;26:1269. doi: 10.1186/s12870-026-09068-7 (PMC13403587; doi:10.1186/s12870-026-09068-7)
Supplement: Supplementary file 1 — Supplementary Material 1. [file 12870_2026_9068_MOESM1_ESM.zip › Supplementary Table S6.docx]

**Supplementary Table S6** Clustering classification of 120 *I. polycarpa* germplasm resources on IRAP markers

| Group | To. | GY | QN | QXN | LPS | ZY | TR | QDN |
| --- | --- | --- | --- | --- | --- | --- | --- | --- |
| Ⅰ | 38 | XW3, XW4 | DY3, GD2, GD7, HS2 | CH1, XR1, XR2, XR3, XY1, XY2, XY3, XY8, XY10, XY11, XY15, XY16, XY17 | DF1, DF2, LPS5, LPS16, LPS17, LPS18, LPS19, LPS20, LPS21, LPS22, LPS23, LPS24, LPS25, LPS26, LPS27 | HC2 | JK3, YJ8, YJ9 |  |
| Ⅱ | 10 |  |  |  | LPS3, LPS4, LPS6, LPS7, LPS9 | HC1, MT1, MT2, MT3, MT4 |  |  |
| Ⅲ | 38 | GY1, GY2, GY3, GY4, XW1, XW2, XW5 | GD1, GD5, GD10 | XY4, XY9, XY14 | LPS1, LPS2, LPS8, LPS10, LPS11, LPS12 | MT5, SY1, SY2, SY3, SY4, SY5, SY6 | JK1, JK2, JK4, ST1, ST2, ST3, YJ2, YJ3, YJ6 | JP1, JP2, JP3 |
| Ⅳ | 34 |  | DY1, DY2, GD3, GD4, GD6, GD8, GD9, GD11, HS1, LB1, LB2 | PA1, PA2, XY5, XY6, XY7, XY12, XY13 | LPS13, LPS14, LPS15 |  | WS1, WS2, YJ1, YJ4, YJ5, YJ7 | DZ1, DZ2, JH1, JH2, LS1, LS2, LS3 |
