# Supplementary material for: SSR and IRAP-based genetic diversity analysis for core collection of Idesia polycarpa
Source: BMC Plant Biol. 2026 May 28;26:1269. doi: 10.1186/s12870-026-09068-7 (PMC13403587; doi:10.1186/s12870-026-09068-7)
Supplement: Supplementary file 1 — Supplementary Material 1. [file 12870_2026_9068_MOESM1_ESM.zip › Supplementary Table S8.docx]

**Supplementary Table S8a** Within-population Jaccard similarity coefficients

| Population | N | Mean similarity | SD | Min | Max |
| --- | --- | --- | --- | --- | --- |
| GY | 9 | 0.313 | 0.137 | 0.121 | 0.591 |
| QN | 18 | 0.309 | 0.136 | 0.105 | 0.552 |
| QXN | 23 | 0.291 | 0.143 | 0.088 | 0.630 |
| LPS | 29 | 0.256 | 0.134 | 0.081 | 0.664 |
| ZY | 13 | 0.317 | 0.112 | 0.128 | 0.540 |
| TR | 18 | 0.285 | 0.121 | 0.100 | 0.552 |
| QDN | 10 | 0.391 | 0.123 | 0.225 | 0.583 |

**Supplementary Table S8b** Between-population mean Jaccard similarity coefficients

|  | GY | QN | QXN | LPS | ZY | TR | QDN |
| --- | --- | --- | --- | --- | --- | --- | --- |
| GY | - | 0.269 | 0.232 | 0.217 | 0.285 | 0.263 | 0.303 |
| QN |  | - | 0.232 | 0.212 | 0.252 | 0.272 | 0.320 |
| QXN |  |  | - | 0.214 | 0.205 | 0.204 | 0.221 |
| LPS |  |  |  | - | 0.224 | 0.212 | 0.214 |
| ZY |  |  |  |  | - | 0.264 | 0.282 |
| TR |  |  |  |  |  | - | 0.294 |
| QDN |  |  |  |  |  |  | - |

Table note: Values are mean Jaccard similarity coefficients. For within‑population, all pairwise comparisons among individuals within each population; for between‑population, all pairwise comparisons between individuals from the two populations.
