# Supplementary figures and images for: SSR and IRAP-based genetic diversity analysis for core collection of Idesia polycarpa
Source: BMC Plant Biol. 2026 May 28;26:1269. doi: 10.1186/s12870-026-09068-7 (PMC13403587; doi:10.1186/s12870-026-09068-7)

Rarefaction curves of gene diversity (H)

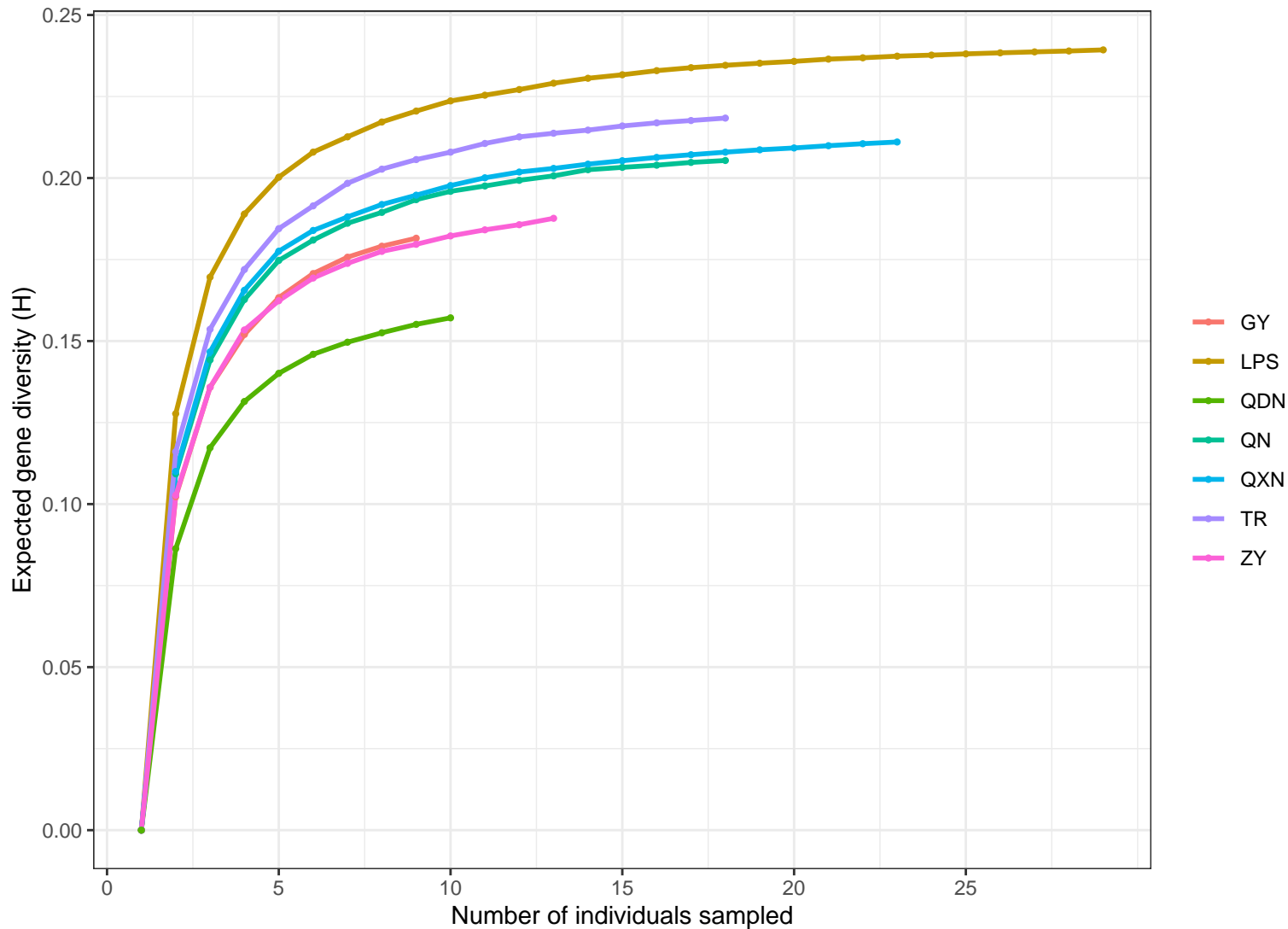

Supplement: Supplementary file 2 — Supplementary Material 2. [file 12870_2026_9068_MOESM2_ESM.zip › Supplementary Fig. S2.pdf]
