# Supplementary material for: SSR and IRAP-based genetic diversity analysis for core collection of Idesia polycarpa
Source: BMC Plant Biol. 2026 May 28;26:1269. doi: 10.1186/s12870-026-09068-7 (PMC13403587; doi:10.1186/s12870-026-09068-7)
Supplement: Supplementary file 2 — Supplementary Material 2. [file Supplementary Fig. S1.pdf › Supplementary Fig. S1.pdf]

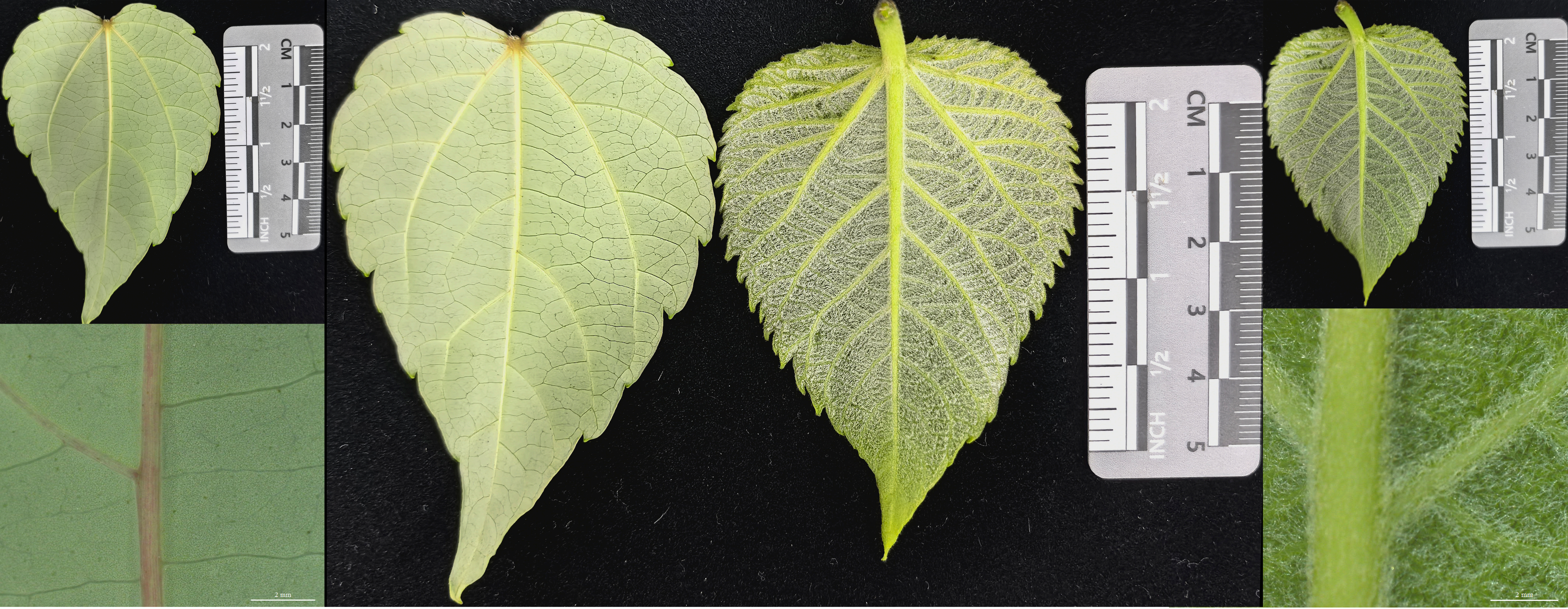

Glabrous **Pubescent**
